# Coronavirus disease 2019 in solid organ transplant recipients in the setting of proactive screening and contact tracing of Qatar

**DOI:** 10.5339/qmj.2021.23

**Published:** 2021-08-10

**Authors:** Rand A. Alattar, Shahd H. Shaar, Muftah Othman, Sulieman H. Abu Jarir, Samar M. Hashim, Fatima Iqbal, Fatima Rustom, Muna A. Almaslamani, Ali S. Omrani

**Affiliations:** ^1^Communicable Diseases Center, Hamad Medical Corporation, Doha, Qatar; ^2^Division of Nephrology, Hamad Medical Corporation, Doha, Qatar; ^3^Division of Infectious Diseases, Hamad Medical Corporation, Doha, Qatar

**Keywords:** coronavirus, SARS-CoV-2, transplantation, middle east, Qatar

## Abstract

Background: Clinical data on Coronavirus Disease 2019 (COVID-19) in solid organ transplant (SOT) recipients are limited. We herein report the initial clinical experience with COVID-19 in SOT recipients in Qatar.

Methods: All SOT recipients with laboratory-confirmed COVID-19 up to May 23, 2020 were included. Demographic and clinical data were extracted retrospectively from the hospital’s electronic health records. Categorical data are presented as frequency and percentages, while continuous variables are summarized as medians and ranges.

Results: Twenty-four SOT recipients with COVID-19 were identified (kidney 16, liver 6, heart 1, and liver and kidney 1). Organ transplantation preceded COVID-19 by a median of 60 months (range 1.7–184). The median age was 57 years (range 24–72), and 9 (37.5%) transplant recipients were females. Five (21%) asymptomatic patients were diagnosed through proactive screening. For the rest, fever (15/19) and cough (13/19) were the most frequent presenting symptoms. Five (20.8%) patients required invasive mechanical ventilation in the intensive care unit (ICU). Eleven (46%) patients developed acute kidney injury, including three in association with drug-drug interactions involving investigational COVID-19 therapies. Maintenance immunosuppressive therapy was modified in 18 (75%) patients, but systemic corticosteroids were not discontinued in any. After a median follow-up of 226 days (26–272), 20 (83.3%) patients had been discharged home, 2 (8.3%) were still hospitalized, 1 (4.2%) was still in the ICU, and 1 (4.2%) had died.

Conclusions: Our results suggest that asymptomatic COVID-19 is possible in SOT recipients and that overall outcomes are not uniformly worse than those in the general population. The results require confirmation in large, international cohorts.

## Introduction

By January 2021, the global number of infections caused by Severe Acute Respiratory Syndrome Coronavirus-2 (SARS-CoV-2), the cause of Coronavirus Disease 2019 (COVID-19), was approaching 90 million, with nearly 2 million associated deaths.^1^ Clinical outcomes associated with COVID-19 appear to be considerably worse in older individuals and in those with chronic medical conditions such as hypertension, diabetes mellitus, and chronic kidney disease.^2^ In general, respiratory viral infections in solid organ transplant (SOT) recipients are associated with more rapidly progressive and severe disease, prolonged viral shedding, and a higher risk of mortality.^3^ However, SOT recipients are under-represented in large COVID-19 cohorts, and the interaction between COVID-19 and SOT has not been fully elucidated.^4^ While the immune-suppressed status of SOT recipients may increase their risk of severe COVID-19 and mortality, it is possible that chronic immune suppression may mitigate some of the severe inflammation-driven COVID-19 manifestations.^5^ We herein report the clinical characteristics, management, and outcomes of SOT-associated COVID-19 in Qatar.

## Materials And Methods

Hamad Medical Corporation encompasses multiple hospital facilities and provides all COVID-19 medical care for the population of Qatar. SARS-CoV-2 infection was diagnosed on respiratory tract specimens by real-time polymerase chain reaction (RT-PCR) assays using the TaqPath COVID-19 Combo Kit (Thermo Fisher Scientific, Waltham, Massachusetts) or the Cobas SARS-CoV-2 Test (Roche Diagnostics, Rotkreuz, Switzerland). SARS-CoV-2 testing was offered to all individuals presenting with symptoms suggestive of COVID-19, known close contacts of confirmed cases including healthcare workers, and all returning travelers.

The Communicable Diseases Center’s COVID-19 database includes records of all RT-PCR-confirmed SARS-CoV-2 infections in Qatar. The database was used to identify all SOT recipients who had a laboratory-confirmed SARS-CoV-2 infection by May 23, 2020. Demographic and clinical data were extracted from the hospital’s electronic health records (Cerner Millennium, Cerner Corporation, Kansas City, Missouri, United States of America) during the period from May 25 to June 21, 2020. Final disposition was based on the patients’ status on December 20, 2020. The outcomes assessed included COVID-19 severity according to the World Health Organization.^2^ Acute kidney injury (AKI) was defined according to the Kidney Disease Improving Global Outcomes (KDIGO) guidelines.^6^

COVID-19 diagnosis was confirmed using real-time polymerase chain reaction (RT-PCR) on airway specimens (Thermo Fisher Scientific, Waltham, Massachusetts). All patients received standard clinical care as per the local COVID-19 management guidelines. In addition to supportive care, individual patients received hydroxychloroquine, azithromycin, oseltamivir, lopinavir/ritonavir, darunavir/cobicistat, and/or tocilizumab. Specific regimens were selected by the treating physicians based on the disease severity and the presence of any organ dysfunction or potential drug-drug interactions. Decisions relating to immunosuppressive therapy were made by the attending transplant teams. Tacrolimus therapeutic drug levels were determined by an electro-chemiluminescence immunoassay (Roche Diagnostics, Rotkreuz, Switzerland) with a target reference range of 8.5–17 ng/mL.

Categorical data are presented as frequencies and percentages, while continuous variables are summarized as medians and ranges. Statistical analyses were performed using Microsoft Excel 2016 (Microsoft Corporation, Redmond, Washington, United States of America). The study protocol is consistent with the ethical guidelines of the 1975 Helsinki Declaration and was approved by the Institutional Review Board with a waiver of informed consent (MRC-01-20-191).

## Results

A total of 24 patients with SOT-associated COVID-19 were included. The majority were males (15, 62.5%), and the median age was 57 years (range 24–72). Organs received included the kidney (16, 66.7%), liver (6, 25%), heart (1, 4.2%) and combined liver and kidney (1, 4.2%). The median time from transplant to COVID-19 diagnosis was 60 months (2–184).

Prior to COVID-19 diagnosis, all patients were independent for activities of daily living. Co-existing diabetes mellitus was present in 17 patients (70.8%), and cardiovascular disease in 4 (16.7%). Maintenance immune suppression therapy consisted of tacrolimus (FK) (21, 87.5%), mycophenolate mofetil (MMF) (21, 87.5%), prednisolone (PRD) (19, 79.2%), and/or cyclosporine (CsA) (3, 12.5%).

Five (20.8%) patients were asymptomatic at the time of COVID-19 diagnosis. Two patients were diagnosed during routine COVID-19 screening on return from travel, and two were screened when several members of their families were diagnosed with COVID-19. The fifth patient was a kidney recipient who was admitted with portal vein thrombosis without fever or respiratory symptoms (Table S1 in the supplement file). For the remaining 19 patients, the median time between symptom onset and hospitalization was four days (1–14). The most frequent presenting symptoms were fever (15, 62.5%), cough (13, 54%), malaise (6, 25%), and dyspnea (6, 25%) (Table 1).

At presentation, median oxygen saturation was 96% (89–100). Median baseline laboratory findings included total peripheral white cell count of 5.4 × 10^9^/L (1.9–8.7), lymphocyte count of 0.8 × 10^9^/L (0.3–2.6), ferritin 539 µg/L (68–1161), and CRP 50.2 mg/L (0.3–203) (Table 1).

Baseline radiological abnormalities consistent with pneumonia were documented in 16 (66.7%) patients. Baseline characteristics of individual patients are summarized in Table S1 in the supplementary file.

All patients received azithromycin. Other investigational therapies used are shown in Table 2. All but six patients had their maintenance immune suppressive therapy modified as part of their COVID-19 management. The most frequent changes were dose reduction or suspension of MMF (17/21, 81%) and tacrolimus (8/21, 38.1%). While systemic corticosteroids were not discontinued in any of the patients who were on maintenance prednisolone, the dose was increased in 12/19 (63.2%) patients, including all five who required invasive mechanical ventilation (Table 2, and Table S2 in the supplementary file).

The most frequently observed complication was AKI (11, 46%), involving eight kidney, two liver, and one heart transplant recipient. Three patients developed AKI in association with high tacrolimus levels (23.3 ng/mL, 30.5 ng/mL, and 18.8 ng/mL) following the initiation of protease inhibitor augmentation as an investigational COVID-19 therapy (Table S2 in the supplementary file). By the end of follow-up, renal function returned to baseline in all three of these patients. One incident of AKI occurred in a patient with congestive heart failure and a concomitant urinary tract infection, and another in a patient who received concomitant trimethoprim-sulfamethoxazole. Both patients had had pre-existing chronic kidney graft dysfunction with estimated glomerular filtration rates below 30 mL/min and were still in hospital at the end of follow-up (Table S2 in the supplementary file). Other complications included acute liver dysfunction (9, 37.5%), none of which occurred in a liver transplant recipient.

Five (20.8%) patients required invasive mechanical ventilation for severe ARDS. Of these, two were successfully extubated and transferred to the medical floors after 24 and 33 days and were eventually discharged home, and one patient died after 26 days of admission. The remaining two patients had developed anoxic brain injury and were still in hospital at the end of follow-up (Table 2 and Table S2 in the supplementary file).

After a median follow-up period of 226 days (26–272), 20 patients (83.3%) had been discharged home or to a community isolation facility, 2 (8.3%) were still hospitalized in non-ICU areas, 1 (4.2 %) was still in ICU, and 1 (4.2%) had died (Table 2).

By end of follow-up, negative SARS-CoV-2 RT-PCR tests on airway samples were documented in 19 (79.2%) patients. Viral clearance in those patients occurred within 5–55 days from the first positive results (Table S2 in the supplementary file).

## Discussion

We herein report the clinical presentation, management, and outcomes of SOT-associated COVID-19 in Qatar. To control the epidemic, local authorities implemented several measures, including travel restrictions, social distancing interventions, and proactive screening of repatriated travelers and contacts of confirmed COVID-19 cases. A notable finding in this report is the proportion of SOT recipients with asymptomatic SARS-CoV-2 infections, which were only detected because of intensive case identification efforts. Asymptomatic COVID-19 cases are very well documented in general, and their role in sustaining the pandemic is becoming increasingly appreciated.^7^ However, to the best of our knowledge, this is the first time that asymptomatic SARS-CoV-2 infections are reported in SOT recipients.

Previous reports had suggested that SOT recipients are at increased risk of severe COVID-19. For example, one multi-center report from New York City included 90 SOT recipients with COVID-19, out of whom 24 (26.7%) required mechanical ventilation, and 16 (17.8%) died.^8^ Another single-center study from Madrid, Spain, described 18 SOT recipients with COVID-19; 4 (22.2%) developed progressive respiratory failure, and 5 (27.8%) died.^9^ On the other hand, in this report, only five patients (20.8%) required invasive mechanical ventilation, of which only one patient (4.2%) died.

The low case fatality rate in this report and the satisfactory overall clinical outcomes cannot be fully explained by patient demographics. Several patients in this series recovered after developing organ failure. Those who required invasive mechanical ventilation were aged 53, 54, 58, 61, and 63 years; in contrast, the asymptomatic patients were aged 24, 44, 55, 62, and 69 years (Table S1 and Table S2 in the supplementary file).

Unlike a recent report of severe COVID-19 in a patient who presented shortly after liver transplantation,^10^ one patient in this study received a liver graft from a deceased donor only seven weeks prior to her diagnosis with asymptomatic SARS-CoV-2 infection (Patient 4 in Table S1 and Table S2). Rather than a simple association between the type of graft and severity of COVID-19, the overall health status of the individual may be the most relevant factor. Large, preferably multi-center, cohort studies are urgently required to inform the risk assessment of SOT recipients with COVID-19 and to guide their medical management.

Despite widespread off-label use of hydroxychloroquine, azithromycin, and lopinavir/ritonavir for COVID-19, none of these agents has been proven clinically effective.^11, 12^ In addition to significant potential adverse events, such as cardiac arrhythmias and liver toxicity, these investigational agents could have detrimental interactions with immunosuppressants.^11–13^ As seen in this study and others, those interactions can result in toxicities that outweigh any potential benefits.^14, 15^ Moreover, it is not yet known if the immune-suppressed status of SOT recipients may ameliorate the inflammatory manifestations of COVID-19. Notably, dexamethasone use is associated with significantly reduced mortality in COVID-19 patients who require oxygen support.^16^ It is not yet clear if such a benefit is likely in SOT recipients who are already on a maintenance immune suppression regimen with or without corticosteroids.^4^

Current recommendations for the management of severe COVID-19 in SOT recipients suggest that calcineurin inhibitors and MMF may be reduced or, if necessary, withheld, but the risk of graft dysfunction and rejection should be weighed very carefully against the risk of progressive COVID-19.^17, 18^ In this series, corticosteroids were either maintained or increased; MMF was continued the same, reduced or stopped; and tacrolimus doses were mostly changed only when potential interactions with investigational agents necessitated doing so. None of our patients experienced any evidence of acute rejection. It appears that a reasonable strategy in SOT recipients with COVID-19 is to maintain safe levels of immunosuppression while avoiding unproven investigational agents.^18^ Clinical studies are urgently required to examine the role of investigational COVID-19 therapies in SOT settings and to help determine the most appropriate immunosuppressive therapy modification to minimize the risk of both severe infection and graft rejection.^17^

Patients in this study who cleared SARS-CoV-2 from their airways did so within as short a time span as 5 days and up to as long as 55 days or more. This is consistent with known variability in SARS-CoV-2 shedding, especially in patients with severe COVID-19.^19^

## Conclusion

In conclusion, we report our experience thus far with COVID-19 in SOT recipients in Qatar. Proactive screening allowed the identification of asymptomatic SARS-CoV-2 infections. While some patients developed severe disease and required ICU support, the majority recovered without long-term complications. One patient died during follow-up. Our findings suggest that SOT-associated COVID-19 is not necessarily associated with a uniformly poor clinical outlook. Large clinical studies are required to better understand the clinical course of COVID-19 in SOT recipients, identify risk factors for severe disease, and determine the most appropriate management strategies.

### Disclosure

The authors declare no conflicts of interest.

### Funding

No funding was required.

### Data Availability

Data to support this report are available on reasonable request from the corresponding author.

## Figures and Tables

**Table S1:** Baseline characteristics of 24 solid organ transplant recipients with SARSCoV2 infection

Case	Age (years)	Sex	Type of SOT	Month and year of transplant	Recent travel	Type of presentation	Date of 1^st ^positive COVID-19 PCR	Lymphocyte count ( × 10^9^/L)	Ferritin (μg/L)	CRP (mg/L)	Radiological findings

1	63	Male	Kidney, deceased donor	June 2014	None	Symptomatic	March 23, 2020	0.5	335	202.8	Bilateral patchy infiltrates

2	72	Female	Heart, deceased donor	July 2007	None	Symptomatic	April 3, 2020	0.7	1161	67.2	Pulmonary congestion and pleural effusion

3	62	Female	Liver, living donor	April 2017	Sri Lanka	Screening	March 30, 2020	1.3	328	0.3	Unremarkable

4	44	Female	Liver, deceased donor	February 2020	United Kingdom	Screening	April 3, 2020	0.6	NA	1.3	Unilateral infiltrates

5	61	Female	Kidney, living donor	May 2015	None	Symptomatic	April 14, 2020	0.7	658	116.4	Bilateral patchy infiltrates

6	40	Male	Kidney, living donor	July 2018	Philippines	Symptomatic	April 20, 2020	1.6	810	15.3	Unremarkable

7	46	Male	Kidney, living donor	July 2015	None	Symptomatic	April 25, 2020	2.6	362	5.0	Bilateral patchy infiltrates

8	40	Male	Kidney, living donor	September 2019	None	Symptomatic	April 30, 2020	0.68	72.9	48.4	Unilateral infiltrates

9	62	Female	Liver, deceased donor	November 2015	None	Symptomatic	May 5, 2020	0.8	530	121	Bilateral patchy infiltrates

10	69	Male	Kidney, living donor	June 2005	None	Screening	May 8, 2020	1.3	NA	52	Unremarkable

11	54	Male	Kidney, deceased donor	October 2015	None	Symptomatic	May 3, 2020	0.77	930	39	Unremarkable

12	58	Male	Kidney, living donor	January 2014	None	Symptomatic	May 4, 2020	0.3	231	28.8	Bilateral patchy infiltrates

13	61	Female	Kidney, living donor	December 2010	None	Symptomatic	May 15, 2020	0.8	1055	46.1	Bilateral patchy infiltrates

14	47	Male	Kidney, deceased donor	January 2016	None	Symptomatic	May 15, 2020	0.8	173	31	Unremarkable

15	52	Male	Kidney, living donor	July 2016	None	Symptomatic	May 15, 2020	1.5	822	97.6	Bilateral patchy infiltrates

16	55	Male	Kidney, living donor	March 2013	None	Screening	May 17, 2020	1.09	68.2	63	Unremarkable

17	72	Male	Liver and kidney, deceased donors	November 2008	None	Symptomatic	May 17, 2020	0.9	925	87.6	Bilateral patchy infiltrates

18	24	Male	Liver, deceased donor	August 2016	None	Screening	May 17, 2020	1.7	97.4	5.0	Unremarkable

19	58	Male	Kidney, living donor	Feb 2008	None	Symptomatic	May 18, 2020	0.6	868.0	113.8	Bilateral patchy infiltrates

20	59	Male	Liver, deceased donor	June 2014	None	Symptomatic	May 19, 2020	1.2	293.0	144.2	Bilateral patchy infiltrates

21	54	Female	Kidney, living donor	Oct 2015	None	Symptomatic	May 20, 2020	1.3	547	21.6	Bilateral patchy infiltrates

22	53	Female	Kidney, living donor	April 2005	None	Symptomatic	May 21, 2020	0.6	693.0	133.0	Bilateral patchy infiltrates

23	39	Male	Liver, deceased donor	Jan 2016	None	Symptomatic	May 21, 2020	0.7	775	5.2	Bilateral patchy infiltrates

24	60	Female	Kidney, living donor	2009^*^	None	Symptomatic	May 23, 2020	1.0		63.1	Bilateral patchy infiltrates


^*^month of transplant is not available. COVID-19, Coronavirus Disease 2019; CRP, C-reactive protein

**Table 1:** Baseline characteristics of 24 solid organ transplant recipients with SARS-CoV-2 infection

Characteristics	All patients (n = 24)

Age, years	57 (24–72)

Female sex	9 (37.5%)

Transplant organ(s)	

Kidney transplant	16 (66.7%)

Liver transplant	6 (25%)

Heart transplant	1 (4.2%)

Kidney and liver transplant	1 (4.2%)

Time since transplant, months	60 (1.7–184)

Living donor	14 (58.3%)

Travel-related COVID-19	3 (12.5%)

Diabetes mellitus	17 (70.8%)

Cardiovascular disease	4 (16.7%)

Charlson Comorbidity Index Score	3 (0–11)

Maintenance immunosuppression	

Tacrolimus	21 (87.5%)

Mycophenolate mofetil	21 (87.5%)

Prednisolone	19 (79.2%)

Cyclosporine	3 (12.5%)

Presenting symptoms	

Asymptomatic	5 (20.8%)

Fever	15 (62.5%)

Cough	13 (54.2%)

Sore throat	4 (16.7%)

Malaise	6 (25%)

Dyspnea	6 (25%)

Nausea and vomiting	3 (12.5%)

Days from symptom onset to hospitalization	4 (1–14)

Baseline assessment	

Oxygen saturation, %	96 (89–100)

White blood cells, × 10^9^ cells/L	5.4 (1.9–8.7)

Absolute neutrophils count, × 10^9^ cells/L	3.7 (1.2–7.2)

Absolute lymphocyte count, × 10^9^ cells/L	0.8 (0.3–2.6)

Ferritin, μg/L	539 (68–1161)

C-reactive protein, mg/L	50.2 (0.3–202.8)

Radiological findings	

Unremarkable	7 (29.2%)

Unilateral patchy infiltrates	2 (8.3%)

Bilateral patchy infiltrates	14 (58.3%)

Pulmonary congestion and pleural effusion	1 (4.2%)

Initial disposition	

Community isolation facility	1 (4.2%)

Hospitalized, non-ICU	16 (66.7%)

Hospitalized, ICU	7 (29.2%)


Figures represent n (%) or median (range). COVID-19, coronavirus disease 2019; ICU, intensive care unit; SARS-CoV-2, Severe Acute Respiratory Syndrome Coronavirus 2

**Table 2:** Management and outcomes of 24 solid organ transplant recipients with SARS-CoV-2 infection

Variable	All patients (n = 24)

Maximum respiratory support	

Ambient air	13 (54.2%)

Non-invasive ventilation	6 (25%)

Invasive ventilation	5 (20.8%)

Antiviral therapy	

Azithromycin	24 (100%)

Hydroxychloroquine	23 (95.8%)

Lopinavir/ritonavir or Darunavir/cobicistat	4 (16.7%)

Ribavirin	1 (4.2%)

Oseltamivir	12 (50%)

Tocilizumab	8 (33.3%)

Immunosuppressive therapy management	

Tacrolimus

Continued the same	13/21 (61.9%)

Dose reduced	4/21 (19%)

Withheld	4/21 (19%)

Mycophenolate mofetil or mycophenolic acid	

Continued the same	4/21 (19%)

Dose reduced	4/21 (19%)

Withheld	13/21 (61.9%)

Prednisolone or methylprednisolone	

Continued the same	7/19 (36.8%)

Dose increased	12/19 (63.2%)

Length of hospital stay, days^*^	20 (4–248)

Duration for follow-up, days^*^	226 (26–272)

Status at end of follow-up	

Home, never admitted	1 (4.2%)

Discharged home	19 (79.2%)

Still in hospital, non-ICU floor	2 (8.3%)

Still in hospital, ICU	1 (4.2%)

Died	1 (4.2%)


Figures represent n (%) or median (range). The denominator is included if it is less than the total study population of 24 individuals. COVID-19, coronavirus disease 2019; ICU, intensive care unit; SARS-CoV-2, Severe Acute Respiratory Syndrome Coronavirus 2 ^*^up to December 20, 2020–including one patient who died on day 26 of follow-up

**Table S2:** Management, complications, and outcomes of 24 solid organ transplant recipients with COVID-19

Case	IST change	Investigational COVID-19 Therapies	Complications	Mechanical ventilation	ICU admission	Last available SARS-CoV2 PCR (result and days from first test)	Outcomes at end of follow-up

1	FK withheld MMF withheld PRD increased	HCQ, AZT, OST, DRV/c, RBV, TCZ	ARDS, AKI (stage 2), liver injury, gastric bleeding	IMV	Yes	negative (23 days)	Hospital discharge

2	FK continued PRD continued	HCQ, AZT, OST	AKI (stage 1), anemia, congestive heart failure, UTI	NIV	Yes	negative (7 days)	Hospital discharge

3	FK continued MMF dose reduced	HCQ, AZT,	None	None	No	negative (5 days)	Hospital discharge

4	FK continued PRD continued	HCQ, AZT,	AKI (stage 1)	None	No	negative (22 days)	Hospital discharge

5	FK withheld MMF withheld PRD dose increased	HCQ, AZT, OST, LPV/r, TCZ	AKI (stage 2), rhabdomyolysis, ARDS, liver injury, anemia	IMV	Yes	negative (43 days)	Hospital discharge

6	FK continued MMF withheld PRD dose increased	HCQ, AZT, OST	Liver injury	None	No	negative (14 days)	Hospital discharge

7	FK dose reduced MMF continued PRD continued	HCQ, AZT, OST	Liver injury	None	No	positive (28 days)	Hospital discharge

8	FK withheld MMF withheld PRD dose increased	HCQ, AZT, OST	AKI (stage 1)	None	No	positive (25 days)	Hospital discharge

9	FK continued MMF continued	HCQ, AZT, OST	None	None	No	negative (14 days)	Hospital discharge

10	FK continued MMF dose reduced PRD continued	HCQ, AZT	None	None	No	negative (14 days)	Hospital discharge

11	FK withheld MMF withheld PRD dose increased	HCQ, AZT, LPV/r, TCZ	ARDS, QTC prolongation, seizures, cardiorespiratory arrest, AKI (stage 3), liver injury, anoxic brain injury	IMV	Yes	negative (48 days)	Medical floor

12	FK continued MMF withheld PRD dose increased	HCQ, AZT, OST, LPV/r, TCZ	ARDS, hypertensive urgency, cardiorespiratory arrest, AKI (stage 2), liver injury, anoxic brain injury	IMV	Yes	negative (29 days)	Medical floor

13	FK dose decreased MMF withheld PRD dose increased	HCQ, AZT, OST, TCZ	Supraventricular tachycardia, AKI (stage 1), liver injury	NIV	Yes	negative (14 days)	Hospital discharge

14	FK continued MMF withheld PRD dose increased	HCQ, AZT, OST	Liver injury	None	No	negative (14 days)	Hospital discharge

15	FK continued MMF withheld PRD dose increased	HCQ, AZT, OST, TCZ	AKI (stage 1)	NIV	No	positive (8 days)	Hospital discharge

16	CsA continued MMF withheld PRD dose increased	HCQ, AZT	Portal vein thrombosis, atrial fibrillation, ischemic cardiomyopathy syncope	None	No	negative (8 days)	Hospital discharge

17	FK continued MMF continued	HCQ, AZT	QTc prolongation	Tracheostomy	ICU	negative (38 days)	ICU

18	FK continued MMF dose reduced PRD continued	AZT	None	None	No	negative (25 days)	Home (no hospital admission)

19	CsA continued MMF withheld PRD dose increased	HCQ, AZT	Elevated myoglobin	None	No	negative (15 days)	Hospital discharge

20	FK continued	HCQ, AZT, OST	Hypoalbuminemia, AKI (stage 1)	None	No	negative (55 days)	Hospital discharge

21	FK continued MMF continued PRD continued	HCQ, AZT	None	NIV	No	positive (7 days)	Hospital discharge

22	FK reduce MMF withheld PRD dose increased	HCQ, AZT, TCZ	ARDS, liver injury, pancreatitis, rhabdomyolysis, AKI (stage 3)	IMV	Yes	positive (22 days)	Died

23	FK dose decreased MMF withheld	HCQ, AZT	Leucopenia	NIV	No	negative (23 days)	Hospital discharge

24	CsA continued MMF decreased PRD dose same	HCQ, AZT, TCZ	None	None	No	negative (21 days)	Hospital discharge


AKI, acute kidney injury; AZT, azithromycin; COVID-19, Coronavirus Disease 2019; CsA, cyclosporine; DRV/c, darunavir/cobicistat; FK, tacrolimus; HCQ, hydroxychloroquine; IMV, invasive mechanical ventilation; IST, immunosuppressive therapy; LPV/r, lopinavir/ritonavir; MMF, mycophenolate mofetil; NIV, noninvasive ventilation; OST, oseltamivir; PRD, prednisolone; RBV, ribavirin; TCZ, tocilizumab: UTI, urinary tract infection
